# Human hippocampal energy metabolism is impaired during cognitive activity in a lipid infusion model of insulin resistance

**DOI:** 10.1002/brb3.124

**Published:** 2013-02-15

**Authors:** Yaso Emmanuel, Lowri E Cochlin, Damian J Tyler, Celeste A de Jager, A David Smith, Kieran Clarke

**Affiliations:** 1 Cardiac Metabolism Research Group Department of Physiology, Anatomy and Genetics, University of OxfordOxford, United Kingdom; 2 Oxford Centre for Clinical Magnetic Resonance Research (OCMR), University of OxfordOxford, United Kingdom; 3 Oxford Project to Investigate Memory and Ageing (OPTIMA) Nuffield Department of Medicine, University of OxfordOxford, United Kingdom

**Keywords:** Brain glucose uptake, diabetes, insulin signaling, neurometabolic coupling

## Abstract

Neuronal glucose uptake was thought to be independent of insulin, being facilitated by glucose transporters GLUT1 and GLUT3, which do not require insulin signaling. However, it is now known that components of the insulin-mediated glucose uptake pathway, including neuronal insulin synthesis and the insulin-dependent glucose transporter GLUT4, are present in brain tissue, particularly in the hippocampus. There is considerable recent evidence that insulin signaling is crucial to optimal hippocampal function. The physiological basis, however, is not clear. We propose that while noninsulin-dependent GLUT1 and GLUT3 transport is adequate for resting needs, the surge in energy use during sustained cognitive activity requires the additional induction of insulin-signaled GLUT4 transport. We studied hippocampal high-energy phosphate metabolism in eight healthy volunteers, using a lipid infusion protocol to inhibit insulin signaling. Contrary to conventional wisdom, it is now known that free fatty acids do cross the blood–brain barrier in significant amounts. Energy metabolism within the hippocampus was assessed during standardized cognitive activity. ^31^Phosphorus magnetic resonance spectroscopy was used to determine the phosphocreatine (PCr)-to-adenosine triphosphate (ATP) ratio. This ratio reflects cellular energy production in relation to concurrent cellular energy expenditure. With lipid infusion, the ratio was significantly reduced during cognitive activity (PCr/ATP 1.0 ± 0.4 compared with 1.4 ± 0.4 before infusion, *P* = 0.01). Without lipid infusion, there was no reduction in the ratio during cognitive activity (PCr/ATP 1.5 ± 0.3 compared with 1.4 ± 0.4, *P* = 0.57). This provides supporting evidence for a physiological role for insulin signaling in facilitating increased neuronal glucose uptake during sustained cognitive activity. Loss of this response, as may occur in type 2 diabetes, would lead to insufficient neuronal energy availability during cognitive activity.

## Introduction

The presence of insulin receptors (IRs) in the brain was demonstrated by Havrankova et al. (1978). One of the major actions of insulin is to promote glucose uptake. Insulin-mediated glucose uptake occurs primarily via the glucose uptake transporter GLUT4 (Mueckler 1994). Animal studies have identified the presence of GLUT4 in the brain colocalizing with the distribution of the IR (Leloup et al. 1996). Subsequent work has demonstrated insulin-stimulated translocation of GLUT4 to the plasma membrane in hippocampal tissue (Reagan 2005; Grillo et al. 2009). The brain is largely dependent on glucose for energy and, unlike peripheral tissue, has a continuous requirement for glucose. Thus, it cannot rely on intermittent postprandial pulses of insulin to stimulate cellular glucose uptake.

IRs and GLUT4 are clearly present in the brain and in vivo human studies using positron emission tomography (PET) have now established that brain glucose uptake is partially sensitive to insulin. Initial work in human healthy volunteers using a hyperinsulinemic euglycemic clamp found no increase in brain glucose uptake during hyperinsulinemia (Hasselbalch et al. 1999). Bingham et al. (2002) subsequently studied the effect of insulin following suppression of basal insulin using somatostatin and found partial increase in glucose metabolism following insulin. Baker et al. (2011) found that in subjects with insulin resistance, there was regional reduction in glucose metabolism and more recently Hirvonen et al. (2011) showed reduced cerebral glucose metabolism in subjects with insulin resistance with improvement following insulin injection. Although insulin is not the major determinant of brain glucose uptake, there is now clear evidence for partial insulin dependence. The precise physiological niche of the insulin-mediated component of brain glucose uptake, however, is not yet clear.

The hippocampus is a vital structure for learning and memory, and IRs have been found in increased density in this region (Havrankova et al. 1978; Unger et al. 1991). There is now growing evidence to support links between neuronal insulin signaling and cognitive function, particularly hippocampal function (McNay and Recknagel 2011; Duarte et al. 2012; Ghasemi et al. 2013). Animal studies have demonstrated increased hippocampal expression of insulin signaling cascade proteins in response to cognitive activity (Zhao et al. 1999; Dou et al. 2005; McNay et al. 2010).

Synaptic neural transmission and molecular changes at the postsynaptic density form the basis for cognitive activity and information storage. Studies in vitro have demonstrated the presence of both IRs and intracellular IR substrate proteins at the postsynaptic density (Abbott et al. 1999). Hori et al. (2005) showed that stimulation of these cells with excitatory neurotransmitters induced rapid accumulation of the IR substrate at postsynaptic sites within minutes. In addition, there is now evidence of de novo synthesis of neuronal insulin (Devaskar et al. 1994). Recently, gene expression for insulin synthesis has been demonstrated in hippocampal neurones and several transcription factors and signaling pathways involved in the development of the pancreas are also active during the formation of the hippocampus (Kuwabara et al. 2011). Furthermore, in vitro studies have also demonstrated local insulin release from synaptosomal preparations in response to local rises in glucose concentration (Santos et al. 1999) and in response to depolarization (Clarke et al. 1986). Considered together, the demonstration of localized neuronal insulin synthesis and excitatory neurotransmitter-induced changes in neuronal insulin signaling capacity (Hori et al. 2005) suggests a close link between neuronal activity, as occurs with cognitive activity, and insulin-signaling-mediated effects on neuronal processes.

As evident in recent published reviews, the effects of insulin in the brain are complex and wide ranging and the possible mechanisms through which insulin may affect memory are multiple (McNay and Recknagel 2011; Duarte et al. 2012; Ghasemi et al. 2013). There is extensive evidence for a metabolic role (indicated by the presence of GLUT4) but also, as these reviews summarize in more detail, evidence for nonmetabolic neuromodulatory effects on synaptic function, neurotransmission, and neurite development, which do not seem to be ostensibly related to any metabolic effects. However, insulin is best known for its role in regulating whole-body metabolism and in the stimulation of glucose uptake in peripheral tissue following postprandial rises in circulating glucose levels. The question therefore arises as to why a hormone that controls metabolic function should also have a role in signaling processes related to cognitive activity. Cognitive function entails an acute increase in energy requirement so intuitively a role for insulin signaling connecting glucose uptake to cognitive function would seem the most parsimonious explanation for the observed links. The basis for the connection is most likely to lie in the specific kinetics of insulin-mediated glucose uptake.

In the brain, glucose uptake occurs primarily via GLUT1 and GLUT3, which are independent of insulin (Mueckler 1994). GLUT1 facilitates a continual basal level of glucose uptake (Vannucci 1994). Following initial uptake across the blood–brain barrier, subsequent uptake from the interstitium into neurones occurs via GLUT3 (Mueckler 1994; Simpson et al. 2007). Compared with plasma glucose concentrations, the interstitial glucose concentration in the brain is relatively low, around 2 mmol/L (Silver and Erecinska 1994). GLUT3 has a low Michaelis constant (*K*_m_), for glucose uptake, around 1.4 mmol/L (Gould et al. 1991; Simpson et al. 2007), so is readily saturated at low glucose concentrations. The GLUT3 transporter therefore operates at near maximal capacity even at low ambient glucose concentrations (Gould et al. 1991). While this allows a steady supply of glucose to the brain, the scope for rapid increase in transport during increased cognitive activity via GLUT3 is limited.

Sustained cognitive activity hugely increases the requirement for glucose and studies in humans confirm increased glucose uptake in association with cognitive activity (Fox et al. 1988; Chen et al. 1993). Invasive animal studies using microdialysis techniques also demonstrate rapid decreases in interstitial glucose concentrations during cognitive activity (McNay et al. 2000). Furthermore, McNay et al. (2010) has also demonstrated that hippocampally mediated spatial memory tasks in rats are limited by glucose availability. Neuronal glucose uptake from the interstitium is primarily mediated via GLUT3 and as outlined earlier, because of the specific kinetics, glucose transporter mechanisms at this step are not believed to be rate limiting. The low *K*_m_, however, suggests that the transporter is readily saturated, so GLUT3 transport may not be rate limiting under resting conditions, but the scope for increasing glucose uptake further may be limited. Increased GLUT3 expression may meet this demand, as has been demonstrated in response to chronic increases in neuronal glucose requirements (Vannucci 1994). However, Choeiri et al. (2005) found that acutely increased demand associated with cognitive activity and training tasks was associated with increased hippocampal GLUT1 expression, thus allowing increased glucose uptake from the circulation to the interstitial fluid. GLUT3, however, did not increase. Therefore, additional transport mechanisms may need to be inducted to meet acute increases in neuronal glucose requirement during cognitive activity.

Insulin-mediated glucose uptake occurs by promoting rapid and substantial translocation of GLUT4 from sequestered intracytoplasmic pools to the plasma membrane (Mueckler 1994) within minutes of insulin binding to the receptor. Insulin signaling therefore provides a mechanism able to rapidly stimulate high-affinity additional glucose uptake. These kinetic characteristics would fit with a role for neuronal insulin signaling in facilitating rapid stimulated glucose uptake according to neuronal activity. This is also consistent with Hori's demonstration of excitatory neurotransmitter-induced insulin signaling cascade protein expression in the postsynaptic density (Hori et al. 2005). We propose that this insulin-stimulated transport may provide a mechanism for meeting the increase in glucose requirement during cognitive activity.

To investigate this link between cognitive impairment and insulin resistance, we applied a lipid infusion model of insulin resistance (Dresner et al. 1999; Roden et al. 1999) to study hippocampal tissue energetics in healthy volunteers.

Tissue energetics were investigated by assessing high-energy phosphate levels using phosphorus magnetic resonance spectroscopy (^31^P MRS). Intracellular energy is produced and transported in the form of adenosine triphosphate (ATP). A reduction in glucose supply would result in reduced ATP production. The ATP molecule is readily converted to phosphocreatine (PCr) to provide an intracellular buffer store, which can be readily hydrolyzed to produce ATP. The PCr/ATP ratio therefore provides an index of cellular energy availability, and a reduction in the PCr/ATP ratio reflects reduced ATP production relative to concurrent energy consumption.

## Aim

Inhibition of the insulin signaling mechanisms that putatively facilitate increased glucose uptake during cognitive activity would lead to relative insufficiency of neuronal energy substrate. This energy deficit would be reflected as a reduction in the hippocampal PCr/ATP ratio. The aim of this study was to determine whether hippocampal energetics during cognitive activity were impaired in a human experimental model of insulin resistance.

## Methods

### Subjects

Twelve healthy volunteers with no history of cardiovascular, endocrine, neurological, or psychiatric disease and normal fasting blood glucose levels were recruited from the University of Oxford. All studies were performed at the Oxford Centre for Clinical Magnetic Resonance Research (OCMR). The study was approved by the Milton Keynes Research Ethics Committee and conducted in accordance with the Declaration of Helsinki with written informed consent obtained from all subjects.

### Study design

**Figure 1:**
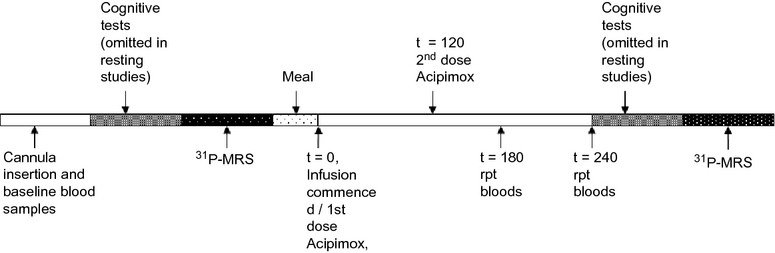
Timeline to show sequence and timing of blood sampling, cognitive testing, and scanning during each study visit.

All subjects were screened prior to entry into the study and were confirmed to have normal fasting glucose levels (<6.0 mmol/L). Subjects were studied over the course of two 1-day visits at least 4 days apart. Subjects arrived in the morning after an overnight fast. A cannula was inserted for blood sampling and for subsequent lipid infusions. Baseline brain energetics during cognitive activity were determined using ^31^P MRS. To stimulate cognitive activity, subjects were asked to perform a set of neuropsychological tests, including two verbal memory tests performed just prior to the scan with the verbal memory delayed recall tasks performed during the scan. Following the baseline assessments, the lipid infusion was commenced for 4 h, after which the tests were repeated. As a control arm, subjects underwent the same assessments, but without the infusions, and instead nicotinic acid tablets were given to prevent the physiological rise in plasma free fatty acid (FFAs) levels that accompany fasting. The order in which the studies were performed was alternated so that half the subjects underwent infusion studies first, and half the subjects had the control arm performed first. Further blood samples were taken at 3 and 4 h after the start of either the infusion or the first dose of nicotinic acid (Fig. 1).

Samples were taken into cold tubes and centrifuged immediately at 2500 rpm at 4°C for 10 min. Plasma was stored at −80°C until analysis. Lipoprotein lipase inhibitor in the form of tetrahydrolipstatin (Xenical, Roche, Welwyn Garden City, U.K.) was added to samples taken for FFA analysis prior to storage to prevent further triglyceride breakdown.

In order to assess whether the lipid infusion itself was associated with changes in resting energetics, a further four subjects were studied using the same protocol, but without cognitive testing. Again, the order of the studies was alternated between subjects.

### Interventions

The lipid infusion protocol to inhibit insulin-mediated glucose uptake was based on published reports showing reduced skeletal muscle cellular glucose uptake and impairment of the insulin signaling cascade (Dresner et al. 1999; Belfort et al. 2005). A triglyceride infusion (20% Intralipid™, Fresenius Kabi, U.K.) was given at 60 mL/h. In order to increase triglyceride breakdown, unfractionated heparin (Monoparin, CP Pharmaceuticals, U.K.) was coadministered at a rate of 0.4 U/kg per min to activate circulating lipoprotein lipase.

To prevent the physiological rise in plasma FFA levels that accompany fasting in the control arm, nicotinic acid (NA) was given as Acipimox 250-mg tablets (Olbetam™, Pharmacia, U.K.) (Chen et al. 1999); one dose following the baseline scan, and a further dose 2 h later.

### Cognitive tests

Executive function, cognitive speed and attention were tested using the Trail making A+B test, the dual tasks test (Baddeley et al. 1997), including the Wechsler Adult Intelligence Scale – Revised (WAIS-R) digit span forwards and backwards (Wechsler 1981), Stroop color word and interference test (Trenerry et al. 1989), and the pattern and letter comparison speed test (Salthouse and Babcock 1991). Visual recall was tested using the Doors B test from the Doors and People test (Thames Valley Testing Company). Verbal recall was tested using the Hopkins Verbal Learning Test – Revised (forms 1, 3, 4, and 6) with delayed recall (Brandt 1991) and the Paragraph Recall Test (Rivermead Behavioral test, Versions A, B, C, and D) (Wilson et al. 1985). The delayed recall parts for both verbal recall tests were performed while the subjects were in the scanner.

### Standardized meal

To prevent the influence of postprandial pulses of insulin, subjects were required to fast for the duration of the visit. In order to limit the physiological responses to prolonged fasting including lipolysis and mild ketosis, a small standardized meal was given following the baseline MRS scan. This was to provide enough carbohydrate to stimulate a small pulse of circulating insulin to prevent ketosis. This small amount of insulin would have dissipated within 2 h, and therefore not interfere with assessments 4 h later. Based on data from Juntunen et al. (2002) in healthy volunteers, a 50-g carbohydrate load, in the form of a slice of bread, stimulated a rise in plasma insulin that returned to fasting levels within 3 h. For this study, a smaller carbohydrate load was given. Subjects were given a waffle, a low-fat yogurt, and a low-sugar fruit juice drink (total carbohydrate 16.3 g, fat 7.3 g, protein 6.2 g). Following this meal, the intervention was started and subjects were kept fasting for the remainder of the study, but were allowed to drink water freely.

### Energetics

The neuropsychological tasks used in this study stimulate hippocampal activation so ^31^P MRS data were acquired to assess hippocampal energetics. Scans were performed using a 3 Tesla system (Siemens Trio, Erlangen, Germany) using a dedicated dual tuned ^1^H/^31^P quadrature birdcage head coil (Rapid Biomedical GmbH, Germany). Proton images for localization and anatomical structural images were obtained using a standard Magnetization Prepared-Rapid Gradient Echo (MP-RAGE) sequence. Spectral data were acquired using a multivoxel acquisition-weighted chemical shift imaging (3D AW-CSI) sequence (Pohmann and von Kienlin 2001). Nuclear overhauser enhancement was employed on all acquisitions to increase signal strength (Li et al. 1996). Automated shimming was performed using the in-built standard Siemens algorithms. Data were acquired from 1024 points, FOV (field of view) 300 mm, TR (repetition time)/TE (echo time) 500/2.3 msec, flip angle 40°, bandwidth 4000 Hz, six averages. In order to maintain signal-to-noise ratio (SNR) and to limit scan time, data were acquired using a 13 × 13 × 13 scan acquisition matrix and data were interpolated for analysis to a 16 × 16 × 16 matrix, giving a nominal voxel size of 6.6 cm^3^ in an acquisition time of 46 min 17 sec. The center of the acquisition grid was positioned at the center of the skull. Data were reconstructed using the Siemens spectroscopy software (Syngo VB13^©^, Siemens, Erlangen, Germany), with a single voxel placed over each hippocampus according to anatomical borders, and summed for each individual. Postprocessing and spectral peak fitting were performed using the AMARES (Vanhamme et al. 1997) algorithm within the jMRUI software package (Naressi et al. 2001) (Version 2.2). Data were corrected for the effects of saturation using the flip angle and T_1_ values (2.39 sec for PCr and 0.79 sec for ATP). Results were confirmed by independent blinded data analysis.

Although a range of stimulation tasks have been developed to be performed while in an magnetic resonance imaging (MRI) scanner, these tasks require the subject to be able to see a projection screen by using MR-compatible mirrors placed over the coils. The design and dimensions of the spectroscopy head coil precluded placement of these mirrors and hence it was not possible to perform these tasks during spectroscopy. To stimulate continued cognitive activity during the spectroscopy acquisition, the delayed recall parts of the verbal recall tasks were performed during the scan. These tasks were performed at the beginning of the CSI acquisition in order to minimize noise in the acquisition from the muscle movements during speech.

Statistical analysis for significance was performed using the two-tailed Student's *t*-test for paired samples with significance taken at *P* < 0.05. The baseline PCr/ATP ratio prior to intervention was averaged for each subject and this averaged value was used as their baseline PCr/ATP ratio for comparison with PCr/ATP ratios after both lipid infusion and NA.

## Results

Eight subjects underwent studies with cognitive activity, but one subject was only able to complete the lipid infusion arm of the cognitive activity studies. Subject characteristics at baseline (Table 1) were the same for those undergoing cognitive testing and those undergoing only resting studies (four subjects).

**Table 1:** Subject characteristics

	Cognitive activity (*n* = 8)	Resting studies (*n* = 4)
Age (years)	25 ± 8	20 ± 0
Gender (M:F)	6:2	2:2
BMI (kg/m^2^)	23 ± 3	21 ± 2

Data expressed as mean ± SD. M, male; F, female; BMI, body mass index.

### Blood tests

For all subjects, baseline fasting insulin, glucose, FFA, and β-hydroxybutyrate were normal (Table 2). The lipid infusion elevated FFA levels from 0.3 ± 0.2 mmol/L at baseline to 1.3 ± 0.3 mmol/L after 3 h and 1.2 ± 0.4 mmol/L after 4 h. During the noninfusion arm, circulating FFA levels decreased. Glucose levels were unchanged over the course of both arms of the study. There was no significant change in insulin levels before and after the lipid infusion. Without infusion, insulin levels after 3 and 4 h were significantly lower than at baseline, 0.23 ± 0.07 mU/L versus 0.17 ± 0.09 mU/L at 3 h, and 0.15 ± 0.09 mU/L at 4 h. β-hydroxybutyrate (B-OHB) values increased with the lipid infusion, 0.39 ± 0.03 mmol/L versus 0.64 ± 0.11 mmol/L at 3 h, and 0.70 ± 0.15 mmol/L at 4 h, but were unchanged over the course of the noninfusion arm (Table 2).

**Table 2:** Blood results

	Lipid infusion				Without lipid infusion			
		
	Glucose (mmol/L)	Insulin (mU/L)	FFA (mmol/L)	B-OHB (mmol/L)	Glucose (mmol/L)	Insulin (mU/L)	FFA (mmol/L)	B-OHB (mmol/L)
Baseline	3.6 ± 0.4	0.21 ± 0.08	0.3 ± 0.2	0.39 ± 0.03	3.5 ± 0.3	0.23 ± 0.07	0.3 ± 0.1	0.38 ± 0.03
3 h	3.5 ± 0.2	0.23 ± 0.11	1.3 ± 0.3**	0.64 ± 0.11**	3.4 ± 0.3	0.17 ± 0.09*	0.1 ± 0.1	0.37 ± 0.02
4 h	3.3 ± 0.3	0.21 ± 0.09	1.2 ± 0.4**	0.70 ± 0.15**	3.4 ± 0.3	0.15 ± 0.09**	0.05 ± 0.05**	0.36 ± 0.01

Data expressed as mean ± SD. Blood results for studies performed both with cognitive testing and resting studies, *n* = 11. B-OHB = β-hydroxybutyrate.

* *P* < 0.001 versus baseline;

** *P* < 0.0001 versus baseline.

### Cognitive tests

Overall performance on cognitive tests was unchanged over both arms of the experiment (Table 3).

**Table 3:** Cognitive test scores

(max score)	HVLT immediate (*n* = 36)	HVLT delayed (*n* = 12)	HVLT DI	Digit span forward	Digit span backward	Doors
Baseline without infusion	30 ± 4	11 ± 1	0 ± 0	8 ± 1	6 ± 1	10 ± 2
Post without infusion	29 ± 4	8 ± 4	0 ± 1	7 ± 1	6 ± 1	11 ± 1
Pre lipid infusion	28 ± 4	10 ± 2	0 ± 1	7 ± 2	6 ± 1	10 ± 2
Post lipid infusion	29 ± 2	9 ± 2	−1 ± 1	8 ± 1	5 ± 1	11 ± 1
	Paragraph recall imm (*n* = 21)	Paragraph recall del (*n* = 21)	Paragraph recall cued	Trails A (sec)	Trails B (sec)	Trails B–A (sec)
Baseline without infusion	13 ± 2	10 ± 2	1 ± 1	20 ± 7	42 ± 21	22 ± 14
Post without infusion	13 ± 3	12 ± 4	1 ± 1	16 ± 3	32 ± 8	16 ± 5
Pre lipid infusion	12 ± 2	9 ± 3	1 ± 1	23 ± 10	55 ± 22	34 ± 21
Post lipid infusion	9 ± 4	8 ± 3	1 ± 1	18 ± 6*	45 ± 26	27 ± 21
	Dual tasks	STROOP CW (*n* = 112)	STROOP CW int (*n* = 112)	Pattern comp	Letter comp	
Baseline without infusion	3.3 ± 1.7	112 ± 0	112 ± 1	17 ± 4	12 ± 2	
Post without infusion	2.5 ± 1.7	112 ± 0	111 ± 1	20 ± 2*	11 ± 2	
Pre lipid infusion	2.6 ± 2.3	112 ± 1	111 ± 1	17 ± 3	10 ± 2	
Post lipid infusion	1.2 ± 1.3	111 ± 2	109 ± 2	18 ± 4	9 ± 2	

Data are expressed as mean ± SD, HVLT, Hopkins Verbal Learning Test; Imm, immediate recall; del, delayed recall; DI, discrimination index; CW, color word; Int, interference; Comp, comparison. Pattern and letter comparison speed score = number correct in 20 seconds.

* *P* = 0.03 vs. baseline, *n* = 7.

### ^31^P Magnetic resonance spectroscopy

**Figure 2:**
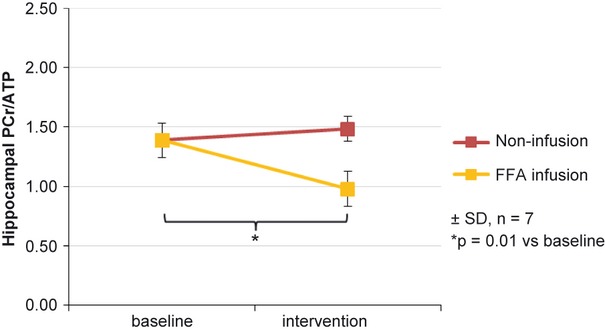
PCr/ATP (phosphocreatine-to-adenosine triphosphate) ratios following cognitive activity. Baseline averaged ratio (1.39 ± 0.40) with drop in ratio after lipid infusion (0.98 ± 0.38, *P* = 0.01 yellow line) but no change following nicotinic acid control study (1.48 ± 0.27, *P* = 0.57, red line).

Data from one subject undergoing studies with lipid infusion and cognitive activity showed significant movement artifact and was therefore excluded from the analysis. The baseline PCr/ATP ratios were the same (1.5 ± 0.6 pre-FFA vs. 1.2 ± 0.4 pre-non-infusion, *P* = 0.8, averaged baseline ratio 1.4 ± 0.4, *n* = 7). In studies performed with cognitive activity and lipid infusion, there was a marked drop in PCr/ATP ratio with cognitive activity following lipid infusion (1.4 ± 0.4 vs. 1.0 ± 0.4, *P* = 0.01, *n* = 7). In the control arm without lipid infusion, PCr/ATP ratios with cognitive activity were unchanged (1.4 ± 0.4 vs. 1.5 ± 0.3, *P* = 0.57, *n* = 7, Fig. 2 and Table 4).

**Table 4:** PCr/ATP ratios with cognitive activity and at rest

	Average baseline	Postlipid infusion	Post without infusion
Cognitive activity (*n* = 7)	1.4 ± 0.4	1.0 ± 0.4 (*P* = 0.01)	1.5 ± 0.3 (*P* = 0.57)
Resting (*n* = 4)	1.5 ± 0.3	1.5 ± 0.6 (*P* = 0.85)	1.3 ± 0.4 (*P* = 0.16)

Data are expressed as mean ± SD. PCr/ATP, phosphocreatine-to-adenosine triphosphate.

In studies performed without cognitive activity in a further four volunteers, the baseline PCr/ATP ratios were the same (1.7 ± 0.3 pre-FFA vs. 1.3 ± 0.1 pre-non-infusion, *P* = 0.1, averaged baseline value 1.5 ± 0.3, *n* = 4). There was no difference in PCr/ATP ratio either after lipid infusion or following no lipid infusion (Table 4).

## Discussion

Cognitive impairments are increasingly recognized in patients with insulin resistance (Elias et al. 1997; Gregg et al. 2000). Loss of this insulin-mediated mechanism for matching glucose uptake to neuronal demand may explain the observed cognitive deficits in tasks of memory, attention, and speed, which all require intense neuronal activity. To date, there have been no experimental studies in healthy human volunteers to determine the dynamic in vivo effects of induced insulin resistance on the brain.

Previous studies of glucose metabolism in the brain have used methods such as ^13^Carbon MRS or PET scans. ^13^C MRS allows assessment of glucose tracking and flux; however, very few centers have the facilities and technical expertise to perform ^13^C MRS in human brain and ^31^P MRS is more widely available. As brain energy metabolism is heavily dependent on glucose, alterations in glucose supply are much more likely to alter overall brain tissue energetics than in peripheral tissue where energy production may be maintained by metabolism of alternative energy substrates. Therefore in the brain, investigation of tissue energetics has the potential to provide sensitive assessment of changes in glucose metabolism resulting from experimental intervention. PET is a well-established tool for studying brain glucose metabolism. However, the radiation risks associated with PET scans, although small, are of concern especially in young healthy volunteers and when carried out repeatedly. ^31^P MRS, unlike PET, does not involve exposure to ionizing radiation and offers a safe and novel approach.

Upon binding of insulin to its receptor, signal transduction begins with activation of the IR substrate complex and subsequent activation of phosphoinositide-3-kinase (PI3-K) (Okada et al. 1994). This leads to translocation of GLUT4 to the plasma membrane (Zierath et al. 1996). McNay et al. (2010) has shown in animal models that local delivery of insulin to the hippocampus results in improved cognitive performance via PI3-K-dependent mechanisms along with increased removal of glucose from the interstitium. Blockade of endogenous hippocampal insulin was found to impair insulin-mediated improvements in cognitive function.

Patients with insulin resistance are known to have increased circulating levels of plasma FFAs (Fraze et al. 1985) and have also been found to have increased brain fatty acid uptake (Karmi et al. 2010). Increases in plasma FFAs using a lipid infusion model have been shown to inhibit insulin signaling via PI3-K-dependent mechanisms (Dresner et al. 1999) and reduce insulin-mediated glucose uptake in skeletal muscle (Dresner et al. 1999; Roden et al. 1999). Lipid infusions and high fat diets have been extensively used to model insulin resistance. Furthermore, contrary to previously held beliefs, there are several recent reports showing that FFAs do in fact cross the blood–brain barrier in significant amounts (Rapoport et al. 2001; Hamilton and Brunaldi 2007; Mitchell et al. 2011). The validity of the model in brain studies is strengthened by McNay et al. (2010) work in animal models demonstrating that insulin resistance, induced using a high-fat diet model, was associated with impaired hippocampal function.

The duration and increase in FFA levels achieved in this study are comparable with previous studies performed in skeletal muscle in which FFA-induced alterations in insulin signaling cascade protein expression were demonstrated on biopsy tissue (Dresner et al. 1999; Roden et al. 1999). In addition, these studies also demonstrated the consequent reduction in whole-body insulin-mediated glucose uptake using hyperinsulinemic–euglycemic clamp techniques, showing reduced glucose infusion requirements following lipid infusion. The standardized meal would have stimulated a small release of peripheral insulin. In the control arm, glucose values were unchanged, but insulin levels were lower after 3 and 4 h compared with baseline values, as may be expected after fasting. However, in the lipid infusion arm, glucose values were no different, but insulin levels did not fall over the course of the study visit. This is consistent with a greater requirement for insulin to maintain euglycemia following the standardized meal, consistent with peripheral insulin resistance. Formal assessment of peripheral insulin resistance, using clamp techniques, was not performed in this study as demonstration of peripheral insulin resistance would not provide direct evidence for neuronal insulin resistance (Dresner et al. 1999; Shulman 2000). It is not possible to obtain hippocampal interstitial FFA levels or tissue biopsy samples from human healthy volunteers to confirm alterations in neuronal insulin signaling. Our experimental design, however, is based on proven models of peripheral insulin resistance (Dresner et al. 1999; Roden et al. 1999) taken together with recent evidence for transport of FFAs across the blood–brain barrier (Rapoport et al. 2001; Hamilton and Brunaldi 2007; Mitchell et al. 2011). The findings are consistent with published work by Karmi et al. (2010) demonstrating increased brain fatty acid uptake in humans with insulin resistance and with McNay et al. (2010) work in animal models demonstrating increases in hippocampal glycolytic rates in response to insulin, and demonstration of impaired cognition in a model of insulin resistance induced by a high-fat diet (McNay et al. 2010).

The cognitive test battery provided stimulation of the cognitive domains in which insulin resistance-associated deficits have been identified and is comparable in task difficulty with the test battery used by Baker et al. (2011). Impairment of the energy supply to sustain this activity would lead to depletion of the intracellular energy stores. The primary purpose of the cognitive testing in this study was to stimulate neuronal activity, and hence the hypothesized stimulated neuronal glucose uptake via insulin signaling. The findings in this study of a reduction in PCr/ATP ratio with cognitive stimulation following lipid infusion to inhibit insulin signaling, and lack of change in the absence of lipid infusion to induce insulin resistance, supports a role for insulin in maintaining neuronal glucose uptake and hence cellular energy production during increased neuronal activity. Observed performance on cognitive testing was not impaired following the lipid infusion, despite the reduction in PCr/ATP ratio. The 20-min cognitive test battery appears to have provided enough stimulation to result in a depletion of intracellular energy stores, and thus test the experimental hypothesis, but the sensitivity of the tests for subtle changes in performance after a brief intervention is limited. Also, it is possible that the PCr energy reserves in these healthy young adult volunteers, although depleted following the lipid infusion, were enough to preserve gross cognitive function during the brief testing period. It is theoretically possible that the reduction in the observed ratio reflects increased ATP consumption in response to lipid infusion, rather than reduced production. The lack of change in PCr/ATP ratio with lipid infusion in the resting studies would suggest that the reduction observed with cognitive activity is more likely to be due to insufficient production relative to demand.

Acetylcholine is an important neurotransmitter and activation of the nicotinic form of the receptor is associated with modulation of neural transmission and beneficial effects on higher brain functions including memory processes (Girod et al. 2000). It is therefore possible that nicotinic acid used in the control arm of the study may have been associated with effects on neuronal processing. However, any putative effects on membrane potentials and transmission processes would require energy and therefore an increased requirement for ATP. If ATP production were unable to meet the extra demand, it would be reflected in a reduced PCr/ATP ratio. However, in the control studies performed before and after nicotinic acid, no differences were seen in PCr/ATP ratios, suggesting that neuronal energy production was sufficient. Nicotinic acid also serves as a precursor for the formation of NAD^+^ (Ross 1998), and hence this may also help to offset any increased energy requirement as a consequence of nicotinic acetylcholine receptor stimulation. The PCr/ATP ratios were unaffected by lipid infusion or nicotinic acid administration in the absence of cognitive activity, implying that resting energetics were unaffected and therefore that resting energy uptake is not affected by insulin. In combination with the observed energetic impairment during cognitive stress, these findings are consistent with the hypothesis that rapid increases in glucose uptake during neuronal activation occur through insulin-mediated mechanisms.

The Randle cycle provides an alternative explanation for lipid-induced reduction in glucose oxidation, whereby increased lipid oxidation results in feedback inhibition of enzymes involved in glycolysis (Randle et al. 1963). This model, however, relates to studies performed in skeletal muscle, which has inherent metabolic flexibility and therefore is capable of using both lipid and glucose. While it is possible that a Randle cycle mechanism may exist in the brain, the metabolic inflexibility of neuronal tissue would suggest that the findings in this study are more likely to be due to changes in insulin-mediated glucose uptake than substrate competition.

In addition to insulin, there is increasing recognition that the hormone leptin may also play an important part in neuronal signaling and cognitive function (Paz-Filho et al. 2010), as well as having a role in homeostasis. Some of these effects are mediated through PI3-K signaling (Donato et al. 2010). It is possible that leptin resistance may contribute to the findings observed in this work; however, a leptin-mediated hypothesis would not explain the presence of the insulin signaling pathway in neuronal tissues and their activation in response to cognitive activity.

Insulin resistance is extremely common and this work demonstrates the impact of short-term insulin resistance on hippocampal energetics in response to mild cognitive activity in healthy volunteers. The application of this model of insulin resistance in the study of brain energetics proposes important new metabolic concepts that may explain the recognized links between insulin resistance and cognitive impairment. The concepts and supporting data demonstrated in this work provide the basis for further work in animal and human models to confirm the precise mechanisms linking cognitive activity with insulin-mediated glucose uptake in the brain.

## Conclusion

It is generally accepted that the belief that the brain is insensitive to insulin is no longer tenable, but the widely held belief that fats do not cross the blood–brain barrier have persisted. Consequently, there have been extremely few studies in the brain that utilize manipulation of fat levels. This study presents a novel application of lipid infusion as a tool to investigate dynamic metabolic mechanisms in the human brain and demonstrates a valuable new in vivo experimental model to investigate insulin resistance in the human brain. Furthermore, this is the first mechanistic study to demonstrate the potential metabolic consequences of experimental insulin resistance in the normal human brain. The findings in this study suggest that insulin signaling plays an important role in matching cognitive activity with the required dynamic increases in glucose uptake in the brain.

## References

[b1] Abbott M-A, Wells DG, Fallon JR (1999). The insulin receptor tyrosine kinase substrate p58/53 and the insulin receptor are components of CNS synapses. J. Neurosci.

[b2] Baddeley A, Della Sala S, Papagno C, Spinnler H (1997). Dual-task performance in dysexecutive and nondysexecutive patients with a frontal lesion. Neuropsychology.

[b3] Baker LD, Cross DJ, Minoshima S, Belongia D, Watson G, Craft S (2011). Insulin resistance and Alzheimer-like reductions in regional cerebral glucose metabolism for cognitively normal adults with prediabetes or early type 2 diabetes. Arch. Neurol.

[b4] Belfort R, Mandarino L, Kashyap S, Wirfel K, Pratipanawatr T, Berria R (2005). Dose-response effect of elevated plasma free fatty acid on insulin signaling. Diabetes.

[b5] Bingham EM, Hopkins D, Smith D, Pernet A, Hallett W, Reed L (2002). The role of insulin in human brain glucose metabolism: an 18fluoro-deoxyglucose positron emission tomography study. Diabetes.

[b6] Brandt J (1991). The Hopkins verbal learning test: development of a new memory test with six equivalent forms. Clin. Neuropsychol.

[b7] Chen W, Novotny EJ, Zhu X, Rothman DL, Shulman RG (1993). Localized 1H NMR measurement of glucose consumption in the human brain during visual stimulation. Proc. Natl. Acad. Sci. USA.

[b8] Chen X, Iqbal N, Boden G (1999). The effects of free fatty acids on gluconeogenesis and glycogenolysis in normal subjects. J. Clin. Invest.

[b9] Choeiri C, Staines W, Miki T, Seino S, Messier C (2005). Glucose transporter plasticity during memory processing. Neuroscience.

[b10] Clarke D, Mudd L, Boyd F, Fields M, Raizada MK (1986). Insulin is released from rat brain neuronal cells in culture. J. Neurochem.

[b11] Devaskar SU, Giddings S, Rajakumar P, Carnaghi L, Menon R, Zahm D (1994). Insulin gene expression and insulin synthesis in mammalian neuronal cells. J. Biol. Chem.

[b12] Donato J, Frazao R, Elias CF (2010). The PI3K signaling pathway mediates the biological effects of leptin. Arg. Bras. Endocrinol. Metabol.

[b13] Dou J-T, Chen M, Dufour F, Alkon DL, Zhao W-Q (2005). Insulin receptor signaling in long-term memory consolidation following spatial learning. Learn. Mem.

[b14] Dresner A, Laurent D, Marcucci M, Griffin M, Dufour S, Cline G (1999). Effects of free fatty acids on glucose transport and IRS-1–associated phosphatidylinositol 3-kinase activity. J. Clin. Invest.

[b15] Duarte AI, Moreira PI, Oliveira CR (2012). Insulin in central nervous system: more than just a peripheral hormone. J. Aging Res.

[b16] Elias P, Elias M, D'Agostino R, Cupples L, Wilson P, Silbershatz H (1997). NIDDM and blood pressure as risk factors for poor cognitive performance. The Framingham study. Diabetes Care.

[b17] Fox P, Raichle M, Mintun M, Dence C (1988). Nonoxidative glucose consumption during focal physiologic neural activity. Science.

[b18] Fraze E, Donner CC, Swislocki AL, Chiou YA, Chen YD, Reaven GM (1985). Ambient plasma free fatty acid concentrations in noninsulin-dependent diabetes mellitus: evidence for insulin resistance. J. Clin. Endocrinol. Metab.

[b19] Ghasemi R, Haeri A, Dargahi L, Mohamed Z, Ahmadiani A (2013). Insulin in the brain: sources, localization and functions. Mol. Neurobiol.

[b20] Girod R, Barazangi N, McGehee D, Role L (2000). Facilitation of glutamatergic neurotransmission by presynaptic nicotinic acetylcholine receptors. Neuropharmacology.

[b21] Gould GW, Thomas HM, Jess TJ, Bell GI (1991). Expression of human glucose transporters in *Xenopus* oocytes: kinetic characterization and substrate specificities of the erythrocyte, liver, and brain isoforms. Biochemistry.

[b22] Gregg EW, Yaffe K, Cauley JA, Rolka DB, Blackwell TL, Narayan KMV (2000). Is diabetes associated with cognitive impairment and cognitive decline among older women?. Arch. Intern. Med.

[b23] Grillo CA, Piroli GG, Hendry RM, Reagan LP (2009). Insulin-stimulated translocation of GLUT4 to the plasma membrane in rat hippocampus is PI3-kinase dependent. Brain Res.

[b24] Hamilton J, Brunaldi K (2007). A model for fatty acid transport into the brain. J. Mol. Neurosci.

[b25] Hasselbalch S, Knudsen G, Videbaek C, Pinborg L, Schmidt J, Holm S (1999). No effect of insulin on glucose blood-brain barrier transport and cerebral metabolism in humans. Diabetes.

[b26] Havrankova J, Roth J, Brownstein M (1978). Insulin receptors are widely distributed in the central nervous system of the rat. Nature.

[b27] Hirvonen J, Virtanen KA, Nummenmaa L, Hannukainen JC, Honka M-J, Bucci M (2011). Effects of insulin on brain glucose metabolism in impaired glucose tolerance. Diabetes.

[b28] Hori K, Yasuda H, Konno D, Maruoka H, Tsumoto T, Sobue K (2005). NMDA receptor-dependent synaptic translocation of insulin receptor substrate p53 via protein kinase C signaling. J. Neurosci.

[b29] Juntunen KS, Niskanen LK, Liukkonen KH, Poutanen KS, Holst JJ, Mykkanen HM (2002). Postprandial glucose, insulin, and incretin responses to grain products in healthy subjects. Am. J. Clin. Nutr.

[b30] Karmi A, Iozzo P, Viljanen A, Hirvonen J, Fielding BA, Virtanen K (2010). Increased brain fatty acid uptake in metabolic syndrome. Diabetes.

[b31] Kuwabara T, Kagalwala MN, Onuma Y, Ito Y, Warashina M, Terashima K (2011). Insulin biosynthesis in neuronal progenitors derived from adult hippocampus and the olfactory bulb. EMBO Mol. Med.

[b32] Leloup C, Arluison M, Kassis N, Lepetit N, Cartier N, Ferre P (1996). Discrete brain areas express the insulin-responsive glucose transporter GLUT4. Mol. Brain Res.

[b33] Li C, Negendank W, Murphy-Boesch J, Padavic-Shaller K, Brown T (1996). Molar quantitation of hepatic metabolites in vivo in proton-decoupled, nuclear overhauser effect enhanced 31P NMR spectra localized by three-dimensional chemical shift imaging. NMR Biomed.

[b34] McNay EC, Recknagel AK (2011). Brain insulin signaling: a key component of cognitive processes and a potential basis for cognitive impairment in type 2 diabetes. Neurobiol. Learn. Mem.

[b35] McNay EC, Fries TM, Gold PE (2000). Decreases in rat extracellular hippocampal glucose concentration associated with cognitive demand during a spatial task. Proc. Natl. Acad. Sci. USA.

[b36] McNay EC, Ong CT, McCrimmon RJ, Cresswell J, Bogan JS, Sherwin RS (2010). Hippocampal memory processes are modulated by insulin and high-fat-induced insulin resistance. Neurobiol. Learn. Mem.

[b37] Mitchell RW, On NH, Miller MR, Del Bigio DW, Hatch GM (2011). Fatty acid transport protein expression in human brain and potential role in fatty acid transport across human brain microvessel endothelial cells. J. Neurochem.

[b38] Mueckler M (1994). Facilitative glucose transporters. FEBS J.

[b39] Naressi A, Couturier C, Castang I, Graveron-Demilly R, de Beer D (2001). Java-based graphical user interface for MRUI, a software package for quantitation of in vivo/medical magnetic resonance spectroscopy signals. Comput. Biol. Med.

[b40] Okada T, Kawano Y, Sakakibara T, Hazeki O, Ui M (1994). Essential role of phosphatidylinositol 3-kinase in insulin-induced glucose transport and antilipolysis in rat adipocytes. Studies with a selective inhibitor wortmannin. J. Biol. Chem.

[b41] Paz-Filho G, Wong ML, Licinio J (2010). The procognitive effects of leptin in the brain and their clinical implications. Int. J. Clin. Pract.

[b42] Pohmann R, von Kienlin M (2001). Accurate phosphorus metabolite images of the human heart by 3D acquisition-weighted CSI. Magn. Reson. Med.

[b43] Randle PJ, Garland PB, Hales CN, Newsholme EA (1963). The glucose fatty-acid cycle, its role in insulin sensitivity and the metabolic disturbances of diabetes mellitus. Lancet.

[b44] Rapoport SI, Chang MCJ, Spector AA (2001). Delivery and turnover of plasma-derived essential PUFAs in mammalian brain. J. Lipid Res.

[b45] Reagan LP (2005). Neuronal insulin signal transduction mechanisms in diabetes phenotypes. Neurobiol. Aging.

[b46] Roden M, Krssak M, Stingl H, Gruber S, Hofer A, Furnsinn C (1999). Rapid impairment of skeletal muscle glucose transport/phosphorylation by free fatty acids in humans. Diabetes.

[b47] Ross SG (1998). Murine glial cells regenerate NAD, after peroxide-induced depletion, using either nicotinic acid, nicotinamide, or quinolinic acid as substrates. J. Neurochem.

[b48] Salthouse T, Babcock R (1991). Decomposing adult age differences in working memory. Dev. Psychol.

[b49] Santos M, Pereira E, Carvaho A (1999). Stimulation of immunoreactive insulin release by glucose in rat brain synaptosomes. Neurochem. Res.

[b50] Shulman GI (2000). Cellular mechanisms of insulin resistance. J. Clin. Invest.

[b51] Silver IA, Erecinska M (1994). Extracellular glucose concentration in mammalian brain: continuous monitoring of changes during increased neuronal activity and upon limitation in oxygen supply in normo-, hypo-, and hyperglycaemic animals. J. Neurosci.

[b52] Simpson IA, Carruthers A, Vannucci SJ (2007). Supply and demand in cerebral energy metabolism: the role of nutrient transporters. J. Cereb. Blood Flow Metab.

[b53] Trenerry M, Crosson B, DeBoe J, Leber W (1989). The Stroop neuropsychological screening test.

[b54] Unger JW, Livingston JN, Moss AM (1991). Insulin receptors in the central nervous system: localization, signalling mechanisms and functional aspects. Prog. Neurobiol.

[b55] Vanhamme L, van den Boogaart A, Van Huffel S (1997). Improved method for accurate and efficient quantification of mrs data with use of prior knowledge. J. Magn. Reson.

[b56] Vannucci SJ (1994). Developmental expression of GLUT1 and GLUT3 glucose transporters in rat brain. J. Neurochem.

[b57] Wechsler D (1981). Manual for the Wechsler adult intelligence scale-revised.

[b58] Wilson B, Cockburn J, Baddeley A (1985). The Rivermead behavioral memory test.

[b59] Zhao W, Chen H, Xu H, Moore E, Meiri N, Quon MJ (1999). Brain insulin receptors and spatial memory. Correlated changes in gene expression, tyrosine phosphorylation, and signaling molecules in the hippocampus of water maze trained rats. J. Biol. Chem.

[b60] Zierath J, He L, Gumà A, Wahlström E, Klip A, Wallberg-Henriksson H (1996). Insulin action on glucose transport and plasma membrane GLUT4 content in skeletal muscle from patients with NIDDM. Diabetologia.

